# Development and psychometric evaluation of the Physical Resilience Instrument for Older Adults (PRIFOR)

**DOI:** 10.1186/s12877-022-02918-7

**Published:** 2022-03-21

**Authors:** Fang-Wen Hu, Cheng-Han Lin, Fang-Ru Yueh, Yu-Tai Lo, Chung-Ying Lin

**Affiliations:** 1 Department of Nursing, College of Medicine, National Cheng Kung University Hospital, National Cheng Kung University, No.138, Sheng-Li Road, Tainan, 70403 Taiwan; 2 Department of Health-Business Administration, Fooyin University, No.151, Jinxue Road, Kaohsiung, 83102 Taiwan; 3 Department of Geriatrics and Gerontology, College of Medicine, National Cheng Kung University Hospital, National Cheng Kung University, No.138, Sheng-Li Road, Tainan, 70403 Taiwan; 4 Institute of Allied Health Sciences, College of Medicine, National Cheng Kung University, No. 1, University Road, Tainan, 70101 Taiwan; 5 Department of Occupational Therapy, College of Medicine, National Cheng Kung University, Tainan, 70101 Taiwan; 6 Department of Public Health, College of Medicine, National Cheng Kung University Hospital, National Cheng Kung University, Tainan, 70101 Taiwan; 7 Biostatistics Consulting Center, College of Medicine, National Cheng Kung University Hospital, National Cheng Kung University, Tainan, 70101 Taiwan

**Keywords:** Adaptation, Factor analysis, Measurement, Rasch analysis, Reliability, Validity

## Abstract

**Background:**

Physical resilience is known to minimize the adverse outcomes of health stressors for older people. However, validated instruments that assess physical resilience in older adults are rare. Therefore, we aimed to validate the Physical Resilience Instrument for Older Adults (PRIFOR) to fill the literature gap.

**Methods:**

Content analysis with content validity was first carried out to generate relevant items assessing physical resilience for older adults, and 19 items were developed. Psychometric evaluation of the 19 items was then tested on 200 older adults (mean [SD] age = 76.4 [6.6] years; 51.0% women) for item properties, factor structure, item fit, internal consistency, criterion-related validity, and known-group validity.

**Results:**

All 19 items had satisfactory item properties, as they were normally distributed (skewness = -1.03 to 0.38; kurtosis = -1.05 to 0.32). However, two items were removed due to substantial ceiling effects. The retained 17 items were embedded in three factors as suggested by the exploratory factor analysis (EFA) results. All items except one had satisfactory item fit statistics in Rasch model; thus, the unidimensionality was supported for the three factors on 16 items. The retained 16 items showed promising properties in known-group validity, criterion-related validity, and internal consistency (α = 0.94).

**Conclusions:**

The 16-item PRIFOR exhibits good psychometric properties. Using this instrument to measure physical resilience would be beneficial to identify factors that could protect older people from negative health consequence. With the use of the PRIFOR, intervention effects could also be evaluated. It is helpful to strengthen resilience and thereby facilitate successful aging.

**Supplementary Information:**

The online version contains supplementary material available at 10.1186/s12877-022-02918-7.

## Introduction

As the length of life and number of older adults keep increasing throughout the world, successful aging encounters the challenges of having sustainable and effectiveness methods. To minimize the adverse outcomes of health stressors that inevitably occur is important for people preventing from function loss and maintaining health across the life span. Indeed, response from an individual to his or her late-life stressors is a key for successful aging [1]. In order to take care of the aforementioned issue, “physical resilience” has been considered to be an important concept. Specifically, physical resilience can be used to understand how an individual’s ability to recover or optimize function in the face of disease or age-related losses [2]. It is defined as “At the whole person level: a characteristic that determines one’s ability to resist or recover from functional decline following health stressors” [3].

People inevitably decline in physical resilience as they aged. Such decline could contribute to the challenges in function maintenance and survival status [4]. Therefore, physical resilience has been proposed to be an important factor to decide whether an older person can achieve successful aging [4]. The National Institute on Aging of the United States announced the essential needs to develop instruments on assessing physical resilience of an individual objectively [1]. Although, the most straightforward way to measure physical resilience is to expose the individual to an experimental stressor. For example, a “stress test” has been proposed as this test could trigger measurable changes in the individual’s parameters of internal equilibrium upon a stressor. Then, how an individual recover from the stressor can be assessed and the capacity of physical resilience can be quantified. Unfortunately, concerns on feasibility and ethical issue of exposing people to experimental stressors should be taken into consideration, especially in old and frail adults. Therefore, using subjective measures such as patient-reported outcomes to assess physical resilience for older adults is a promising alternative.

## Background

Whitson et al. conducted a systematic review and proposed a conceptual model of physical resilience. Physical resilience is influenced by psychosocial factors, genetics, physiological reserve, life experiences, and environment. This conceptual model proposes three possible approaches in quantifying physical resilience, included: (1) phenotype of the individuals (frail, robust, and fatigability); (2) discrepancy in age among individuals (biological age versus chronological age); and (3) the outlined trajectory (resilient or resistant). Finally, physical resilience is influenced by stressors or natural stimuli produced by the abovementioned factors and results in a specific outcome [3]. Of noted, different measurement approaches of physical resilience may tackle different aspects of physical resilience depending on an investigator’s objectives and available data. Colón-Emeric et al. further described two approaches to quantify physical resilience in older adults: a recovery phenotype and an expected recovery differential approach. Recovery phenotype approach describes how quickly and completely a patient recover; it is likely to be useful in clinical applications such as clinical prediction models or outcome classification for intervention research [5]. On the other hand, expected recovery approach quantifies how patients’ actual outcomes are compared to their predicted outcome differentials; it may be better suited to identify mechanisms underlying physical resilience and serve as targets for interventions designed to improve physical resilience [5]. Both approaches require repeated measures of clinical outcomes over time to quantify physical resilience. Therefore, measuring the characteristic physical resilience and development of intervention strategies remains challengeable.

Chhetri et al. further revised a conceptual model of physical resilience and proposed the pre-determinants of physical resilience, including: age, psychosocial factors, health behaviors, genetics, and diseases [6]. They also hypothesized that the impact of these factors on physical resilience is mediated through the intrinsic capacity of the individuals. Intrinsic capacity can be viewed as a high-level integrated measure of physiologic reserve and hence, it can serve as a determinant of physical resilience from multiple-domain measurements (ie, activities of daily living, Geriatric Depression Scale, and Short Portable Mental Status Questionnaire) [6].

Although no gold standard exists for the measurement of physical resilience, the Physical Resilience Scale (PRS) developed by Resnick et al. is the only measurement named with *physical resilience*. Participants respond “agree” or “disagree” as a nominal scale, and higher scores indicate higher physical resilience in the PRS. Moreover, the PRS is consisted of 17 items in a single dimension related to recovery after physical difficulties [7]. However, the PRS was developed to include characteristics known to be associated with successful physical aging, such as humor, social support, adaptability, and capitalizing on one’s strengths; it has a strong focus on psychological resilience. Moreover, based on the physical resilience, it can be detected only when confronted with a stressor. Some participants in the Resnick et al. study [7] denied a recent challenging stressor which limited the reliability and validity of the PRS. Moreover, Wu et al. developed a novel approach for quantifying and classifying physical resilience in a cohort of well-functioning white and black older adults [8]. The resilience measure as the residual taken from the linear model regressing frailty on age, sex, race/ethnicity, a variety of diseases, self-reported health, and number of medications. Participants were classified into three groups—adapters, expected agers, and premature frailer—based on residuals. However, the participants were well-functioning and did not suffer from stressor; it is a major limitation of the aforementioned novel approach [8].

In response to the need for appropriate physical resilience assessment, the development of new instruments to measure an individual’s physical resilience level has been deemed crucial. This study had the purpose of developing and testing a new measure, named as the Physical Resilience Instrument for Older Adults (PRIFOR). The items on the PRIFOR were based on earlier qualitative work regarding how older adults identified their physical stressor [9]. Specifically, we assessed the psychometric properties of this measure by examining its internal consistency, known-group validity, and criterion-related validity.

## Materials and methods

### Item generation

With the initiation of a qualitative approach, an interview guideline was based on the previous research [7, 10] and developed through panel discussions with researchers’ expertise in geriatric medicine and gerontological nursing. The guideline focused on determining the experience of physical resilience of hospitalized older adults, while subsequent questions gradually delved deeper into the management strategies adopted by the patients during hospitalization, and up to 1 month after discharge. A total of 16 participants were enrolled from August to December 2019 and face to face, semi-structured, individual interviews were conducted by researcher in private room to allow them to relax. The first interview was conducted during the hospitalization period and the second was conducted during 1 month after discharge. All interviews were recorded using a voice recorder, and the same researcher conducted all 32 interviews. The detailed information can be obtained elsewhere [9]. The audio-recorded interviews were transcribed verbatim manually to traditional Chinese within 24 h post-interview and count-checked against the digital recorder. Two researchers independently analyzed the transcripts using qualitative content analysis, and disagreements were resolved through discussion or by consulting a third researcher until full agreement was reached. The researchers immersed themselves in the transcripts and identified a list of data-driven codes. Codes were compared and grouped into subcategories, which were then abstracted as generic categories. Finally, the generic categories were grouped into main categories.

We formulated a 20-item questionnaire based on the previous qualitative study [9] and major themes identified in participant interviews (Table 1). Content validity was analyzed using the evaluations from three experts who were experts in geriatric care. They independently assessed the consistency between initial pool items and the construct of physical resilience. Among panel discussion, the item N6 was removed due to redundancy and agreement of the experts; specifically, items N6 and N16 had the strongest correlation coefficients, which is over 0.9. Finally, a 19-item questionnaire was developed (Supplementary Material), the content validity index (CVI) of which was 0.88. After the qualitative study procedures mentioned above, the present study decided an initial version of the PRIFOR to be a data collection tool in psychometric testing.

**Table 1:** Items considered to be indicators of physical resilience

Item #	Item description	Measured dimension	Decision
N1	I am able to recover from illness or injury in the expected duration	Positive thinking	
N2	I believe I can recover from every illness or injury	Positive thinking	
N3	I try to look on the bright side when I am facing illness or injury	Positive thinking	
N4	I focus on my remaining abilities, not on what I cannot do	Positive thinking	
N5	I feel energetic most of the time to do what I have to do		Removed due to EFA results
N6	I can cope with the change in my life after illness or injury	Cope and adjust lifestyle	
N7	I adjust my way of life after illness or injury	Cope and adjust lifestyle	
N8	When I am ill or injured, I accept help from my families and friends	Cope and adjust lifestyle	
N9	When I am ill or injured, I accept medical suggestions from healthcare professionals		Removed due to ceiling effects
N10	When I cannot solve a problem, I know where to find help		
N11	When I need to, I can find someone to help	Cope and adjust lifestyle	
N12	I believe I can handle my daily activities	Cope and adjust lifestyle	
N13	I believe I can recover to do my daily activities after illness or injury	Cope and adjust lifestyle	
N14	No matter the good or bad things, I believe most of them happen for a reason	Belief and hopeful mindset	
N15	Past experience gives me confidence to face new challenges and difficulties	Belief and hopeful mindset	
N16	I accept change in my life after illness or injury		Removed due to redundancy
N17	I am a strong person when I am facing illness or injury	Belief and hopeful mindset	
N18	I expect and plan for my future life	Belief and hopeful mindset	
N19	I can deal with unpleasant or painful feelings like sadness, fear, and anger	Belief and hopeful mindset	
N20	I feel I can handle my life	Belief and hopeful mindset	

*Abbreviations*: EFA Exploratory factor analysis

### Psychometric testing of the generated items on the PRIFOR

#### Sample

The study was approved by the Institutional Review Board of medical center with the registered number of IRB No. B-ER-108–064. The target participants were enrolled from medical wards of a 1,343-bed tertiary-care medical center in southern Taiwan. The researcher visited the study wards Monday through Friday during the study period to recruit eligible patients. Inclusion criteria of the eligibility were (i) participants were age 65 and over, (ii) they had the ability to communicate independently, and (iii) their scores in the Clinical Frailty Scale (CFS) were between 4 and 6 [11]. Written informed consent was obtained for most participants. However, if a patient was unable to sign, a proxy’s consent was obtained. One day was reserved for patients to consider their willingness to participate. The participants were excluded if their admissions were due to the following situations: (i) needing hospice care; (ii) needing surgery; and (iii) needing intensive care. The sample size was calcuated with the consideration of exploratory factor analysis (EFA), the main statistical analysis in the present study. A rule of thumb in deciding sample size for EFA is based on item-participatn ratio, and a ratio of 10 (i.e., one item needs to have 10 participants) is suggested [12]. Given that the first version of PRIFOR (ie, the items generated after qualitative method) contains 20 items, a sample size of 200 is recommended. The size of 200 also fit with the consensus in this field [13]. With the use of 200 participants, the power of testing PRIFOR in the EFA with an oblique rotation method is between 0.80 (when *n* = 100) and 0.98 (when *n* = 300) as evidenced by a prior methodological study [14].

#### Measures

During the interview, participants completed the PRIFOR, the EuroQoL 5-dimension Questionnaire (EQ5D), the Katz Index of Independence in Activities of Daily Living (Katz ADL), the 5-item Geriatric Depression Scale (GDS-5), and the Short Portable Mental Status Questionnaire (SPMSQ).

The PRIFOR includes 19 items rated on 5-point Likert scale (1 = strongly disagree; 5 = strongly agree) assessing aspects of resilience associated with recovery following acute health stressors. The score range for the PRIFOR was between 19 and 95, where a higher score indicating greater levels of physical resilience.

The GDS-5 assesses the depression level of older adults. The scores of the five items are totaled, and a higher score indicates a higher level of depression [15, 16]. The GDS-5 has good psychometric properties, including a negative likelihood ratio of 0.07, a positive likelihood ratio of 4.92, a negative predictive value of 0.94, a positive predictive value of 0.81, a sensitivity of 0.94, and a specificity of 0.81 [17]. The GDS-5 in the present study also had good internal consistency (α = 0.75).

The SPMSQ uses 10 questions to assess how well the cognitive function is for older adults, and a point was given when the older adults provide a wrong answer. An older adult has intact cognitive if answering with fewer than 2 wrong answers; mild cognitive impairment with 3 to 4 wrong answers; moderate cognitive impairment with 5 to 7 wrong answers; and severe cognitive impairment with more than 8 wrong answers. Moreover, educational level is considered for adjusting the SPMSQ score [18]. The SPMSQ Chinese version has good internal consistency (α = 0.70) [19]. Criterion-related validity of the SPMSQ was supported [20]. The SPMSQ in the present study had good internal consistency (α = 0.88).

The Katz ADL determines the function levels of ADL by measuring daily activities of use of incontinence materials, eating, getting up out of a chair, visiting the toilet, dressing, and bathing. Scores between 0 and 2 are given to each daily activity (0 = dependence, 1 = limited assistance, and 2 = independence), and a total score between 0 and 12 can be calculated. Moreover, a higher score indicates better level of ADL independence [21]. The internal consistency of the Katz ADL was satisfactory (α = 0.84–0.94) [22], and the present study also had good internal consistency (α = 0.86).

The EQ5D assesses the quality of life for the general population. It contains five self-administered items with three different descriptions for each item. The three descriptions reflect three levels of health status and are coded as 1 (indicating no problems in health), 2 (indicating moderate problems in health), and 3 (indicating extreme problems in health) [23]. The descriptions in the five items were then converted into a 0–1 scale using the equation generated from a time trade-off technique [24]; a higher score in the 0–1 scale indicates better quality of life. The EQ5D in the present study had good internal consistency (α = 0.72).

#### Data analysis

The descriptive statistics was used to analyze the participant’s demographics data, including mean and frequency. Afterwards, the 19 items in the PRIFOR were analyzed to understand their response distribution. At this stage, items without normally distributed responses (ie, skewness > 3 or kurtosis > 8) [25, 26] and those with an extremely high ceiling or floor effect (ie, > 50%) were removed [27]. The items with normally distributed responses and acceptable ceiling/floor effect were additionally analyzed using the EFA to examine the factor structure of the PRIFOR. The EFA applied the extraction method of principal axis factoring and an oblique rotation (ie, oblimin) to test the PRIFOR factor structure. A factor is extracted when an eigenvalue is larger than 1. After the EFA recommended the factor structure, Rasch analysis with partial credit model was used to reexamine the unidimensionality of each factor recommended by the EFA. More specifically, two types of mean square (MnSq), infit MnSq and outfit MnSq, were applied to examine whether an item fits in its embedded construct. Acceptable infit and outfit MnSq are within the range between 0.5 and 1.5 [28]. If an item has misfit MnSq (either in infit or outfit MnSq), the item is removed and the EFA and Rasch analysis are reanalyzed until satisfactory Rasch fit statistics are achieved.

After the factor structure and the unidimensionality of each factor were verified, Cronbach’s α, Rasch separation reliability, and Rasch separation index were applied to understand the PRIFOR’s scale properties. A value higher than 0.7 in Cronbach’s α and Rasch separation reliability indicates good internal consistency of the PRIFOR. A value higher than 2 in the Rasch separation index indicates good discrimination of the PRIFOR (ie, the PRIFOR can effectively distinguish participants with different levels of physical resilience) [29].

Criterion-related validity was then carried out to understand whether the PRIFOR links well with relevant health-related outcomes. Pearson correlation was used to examine the bivariate correlations between the PRIFOR and each of the following health outcomes: depression (assessed using GDS-5), cognitive function (assessed using SPMSQ), ADL (assessed using Katz ADL), and quality of life (assessed using EQ5D). Except for the correlation with depression, the PRIFOR was expected to have positive correlations with all the health outcomes.

Finally, known-group validity was tested for the PRIFOR using the different levels of frailty among the participants. The participants were first classified into two levels of frailty: vulnerable (ie, scores 4 in the CFS) and mildly to moderately frail (ie, scores 5 to 6 in the CFS). Then, independent t-tests were used to examine whether the PRIFOR can significantly distinguish the participants into the two levels of frailty. Additionally, Cohen’s d (0.2 indicates small effect; 0.5 moderate effect; and 0.8 large effect) [30] was used to understand the effects in distinguishing the two levels of frailty.

## Results

### Participant characteristics

The mean age of the 200 participants was 76.4 years (SD = 6.6), and 51.0% were women. The majority (70.0%) had an educational level lower than elementary school, and approximately three quarters (73.0%) were married.

### Response distribution of the PRIFOR

The response distributions of the 19 items in the newly developed PRIFOR are presented in Table 2. More specifically, all 19 items had satisfactory skewness (-1.03 to 0.38) and kurtosis (-1.05 to 0.32). In addition, the item means were between 3.11 and 4.31, and the information implies that the participants tended to answer a high score. Scrutinizing the frequencies of each response for each item, two items (ie, Item N9 “*when I am ill or injured, I accept medical suggestions from healthcare professionals*” and Item N10 “*when I cannot solve the problem, I know where to find help*”) had substantial ceiling effects. Therefore, the two items were removed for further analyses.

**Table 2:** Response distribution of the physical resilience instrument for older adults (PRIFOR)

Item #	M (SD)	Skewness	Kurtosis	n (%) for responses				
				1	2	3	4	5
N1	3.11 (0.79)	0.38	-0.19	0 (0.0)	43 (21.5)	102 (51.0)	46 (23.0)	9 (4.5)
N2	3.12 (0.80)	0.38	-0.27	0 (0.0)	44 (22.0)	99 (49.5)	47 (23.5)	10 (5.0)
N3	3.30 (0.80)	0.24	-0.02	1 (0.5)	24 (12.0)	103 (51.5)	57 (28.5)	15 (7.5)
N4	3.26 (0.73)	0.20	0.32	1 (0.5)	21 (10.5)	112 (56.0)	57 (28.5)	9 (4.5)
N5	3.32 (0.81)	0.03	-0.25	1 (0.5)	27 (13.5)	91 (45.5)	68 (34.0)	13 (6.5)
N6	3.68 (0.86)	0.05	-0.79	0 (0.0)	13 (6.5)	77 (38.5)	71 (35.5)	39 (19.5)
N7	3.77 (0.88)	-0.11	-0.85	0 (0.0)	13 (6.5)	67 (33.5)	73 (36.5)	47 (23.5)
N8	4.23 (0.84)	-0.62	-0.87	0 (0.0)	3 (1.5)	43 (21.5)	58 (29.0)	96 (48.0)
N9	4.31 (0.93)	-1.03	-0.03	1 (0.5)	6 (3.0)	41 (20.5)	34 (17.0)	118 (59.0)
N10	4.16 (0.99)	-0.74	-0.63	1 (0.5)	9 (4.5)	50 (25.0)	37 (18.5)	103 (51.5)
N11	4.03 (0.92)	-0.41	-0.81	1 (0.5)	5 (2.5)	60 (30.0)	55 (27.5)	79 (39.5)
N12	3.65 (1.13)	-0.26	-0.11	3 (1.5)	35 (17.5)	52 (26.0)	49 (24.5)	61 (30.5)
N13	3.48 (1.20)	-0.04	-1.37	3 (1.5)	52 (26.0)	48 (24.0)	39 (19.5)	58 (29.0)
N14	3.32 (0.87)	0.14	-0.43	1 (0.5)	32 (16.0)	88 (44.0)	61 (30.5)	18 (9.0)
N15	3.18 (0.93)	0.08	-0.48	4 (2.0)	44 (22.0)	80 (40.0)	56 (28.0)	16 (8.0)
N17	3.74 (1.00)	-0.09	-1.05	1 (0.5)	17 (8.5)	76 (38.0)	46 (23.0)	60 (30.0)
N18	3.22 (0.89)	0.11	-0.47	2 (1.0)	40 (20.0)	84 (42.0)	59 (29.5)	15 (7.5)
N19	3.35 (0.81)	0.17	-0.15	1 (0.5)	23 (11.5)	97 (48.5)	62 (31.0)	17 (8.5)
N20	3.19 (0.94)	0.12	-0.62	3 (1.5)	47 (23.5)	76 (38.0)	57 (28.5)	17 (8.5)

### Results of EFA and Rasch analysis

The EFA for the remaining 17 items suggested a three-factor solution (Table 3), of which Items N1–N5 were in the same construct (eigenvalue = 8.82; factor loading = 0.65 to 0.88); Items N6 to N8 with Items N11–N13 were in the same construct (eigenvalue = 1.91; factor loading = 0.54 to 0.81); and Items N14–N19 were in the same construct (eigenvalue = 1.42; factor loading = 0.46–0.94). The Rasch analysis supported the unidimensionality of each factor recommended by the EFA, except for a misfit item (ie, Item N5 “*I feel energetic most of the time to do what I have to do*”; infit MnSq = 1.60, and outfit MnSq = 1.44). The item was then removed and both the EFA and Rasch analysis were reanalyzed for the remaining 16 items.

**Table 3:** Item properties of the physical resilience instrument for older adults (PRIFOR)

Item #	Factor loading			Item-total correlation	Difficulty	Infit MnSq	Outfit MnSq
	Factor 1	Factor 2	Factor 3				
N1	0.83			0.85	0.86	0.82	0.73
N2	0.80			0.85	0.77	0.87	0.74
N3	0.90			0.83	-1.03	0.98	0.79
N4	0.76			0.79	-0.59	1.16	1.03
N6		0.56		0.68	0.40	0.91	0.92
N7		0.61		0.70	0.14	0.88	0.90
N8		0.72		0.64	-1.32	1.08	1.12
N11		0.82		0.70	-0.64	0.98	1.04
N12		0.72		0.75	0.48	1.08	1.04
N13		0.80		0.81	0.94	0.97	0.96
N14			0.46	0.69	0.07	1.13	1.12
N15			0.45	0.74	0.58	1.06	1.04
N17			0.61	0.67	-1.52	1.45	1.43
N18			0.87	0.80	0.41	0.76	0.74
N19			0.95	0.78	-0.08	0.72	0.71
N20			0.86	0.81	0.54	0.83	0.81

Factor 1 = positive thinking, Factor 2 = cope and adjust lifestyle, Factor 3 = belief and hopeful mindset

*Abbreviations*: *MnSq* mean square

The reanalyzed results indicated that Items N1–N4 embedded in the same construct with satisfactory factor loadings and Rasch fit statistics (eigenvalue = 8.34); Items N6–N8 with Items N11–N13 embedded in the same construct with satisfactory factor loadings and Rasch fit statistics (eigenvalue = 1.90); and Items N14–N19 embedded in the same construct with satisfactory factor loadings and Rasch fit statistics (eigenvalue = 1.32). The three constructs were named as “*positive thinking*,” “*cope and adjust lifestyle*,” and “*belief and hopeful mindset*” according to the items embedded in them. The three constructs further demonstrated satisfactory internal consistency (α = 0.93 for “*positive thinking*,” 0.89 for “*cope and adjust lifestyle*,” and 0.91 for “*belief and hopeful mindset*”) and item-total correlations (Table 3). Moreover, the 16-item PRIFOR had excellent internal consistency (α = 0.94). The separation reliability and separation index generated by the Rasch analysis also revealed satisfactory properties for the three constructs (person separation reliability = 0.83 for “*positive thinking*,” 0.83 for “*cope and adjust lifestyle*,” and 0.89 for “*belief and hopeful mindset*”; person separation index = 2.24 for “*positive thinking*,” 2.19 for “*cope and adjust lifestyle*,”, and 2.86 for “*belief and hopeful mindset*”; item separation reliability = 0.93 for “*positive thinking*,” 0.97 for “*cope and adjust lifestyle*,” and 0.96 for “*belief and hopeful mindset*”; item separation index = 3.58 for “*positive thinking*,” 6.04 for “*copy and adjust lifestyle*,” and 4.86 for “*belief and hopeful mindset*”).

### Results of criterion-related and known-group validity

The criterion-related validity of the PRIFOR was supported as the physical resilience assessed using PRIFOR was negatively associated with depression (*r* = -0.25 to -0.39; *p* < 0.001) and positively associated with cognitive function (*r* = 0.17 to 0.32; *p* = 0.02 to < 0.001), ADL (*r* = 0.32 to 0.43; *p* < 0.001), and quality of life (*r* = 0.39 to 0.51; *p* < 0.001) (Table 4). The known-group validity of the PRIFOR was supported by the significantly higher scores with moderate-to-large effects among vulnerable participants (scored 4 in the CFS) than mildly to moderately frail participants (scores 5 and 6 in the CFS): Cohen’s d = 0.70 for PRIFOR “*positive thinking*,” 0.87 for “*cope and adjust lifestyle*,” 0.64 for “*belief and hopeful mindset*,” and 0.89 for the entire PRIFOR (Table 5).

**Table 4:** Criterion-related validity of the physical resilience instrument for older adults (PRIFOR)

	r (*p*-value)			
	PRIFOR_F1	PRIFOR_F2	PRIFOR_F3	PRIFOR_Total
PRIFOR_F1	–			
PRIFOR_F2	0.54 (< 0.001)	–		
PRIFOR_F3	0.66 (< 0.001)	0.61 (< 0.001)	–	
PRIFOR_Total	0.80 (< 0.001)	0.87 (< 0.001)	0.89 (< 0.001)	–
GDS-5	-0.39 (< 0.001)	-0.25 (< 0.001)	-0.38 (< 0.001)	-0.38 (< 0.001)
SPMSQ	0.17 (0.02)	0.32 (< 0.001)	0.21 (0.003)	0.28 (< 0.001)
Katz ADL	0.32 (< 0.001)	0.43 (< 0.001)	0.32 (< 0.001)	0.42 (< 0.001)
EQ5D	0.42 (< 0.001)	0.49 (< 0.001)	0.39 (< 0.001)	0.51 (< 0.001)

F1 = positive thinking, F2 = cope and adjust lifestyle, F3 = belief and hopeful mindset

*Abbreviations*: *GDS-5* 5-item Geriatric Depression Scale, *SPMSQ* Short Portable Mental Status Questionnaire, *Katz ADL* Katz Index of Independence in Activities of Daily Living, *EQ5D* EuroQoL 5-dimension Questionnaire

**Table 5:** Known-group validity of the physical resilience instrument for older adults (PRIFOR), using the clinical frailty scale (CFS)

	Mean (SD)	Mean (SD)	t (*p*-value)	Cohen’s d
	CFS Score 4 (*n* = 98)	CFS Scores 5 and 6 (*n* = 102)		
PRIFOR_F1	3.44 (0.69)	2.97 (0.65)	4.99 (< 0.001)	0.70
PRIFOR_F2	4.13 (0.73)	3.50 (0.72)	6.14 (< 0.001)	0.87
PRIFOR_F3	3.57 (0.74)	3.11 (0.70)	4.54 (< 0.001)	0.64
PRIFOR_Total	3.75 (0.60)	3.22 (0.59)	6.26 (< 0.001)	0.89

F1 = positive thinking, F2 = cope and adjust lifestyle, F3 = belief and hopeful mindset

## Discussion

The PRIFOR was developed and examined for its initial psychometric properties in the present study. We have demonstrated good internal consistency, known-group validity, construct validity, and criterion-related validity for this newly developed questionnaire.

The response distribution of the PRIFOR indicated that the two items had substantial ceiling effects. It is possible, for example, that all participants admitted to the medical wards had been seeking and accepting help from healthcare professionals. Thus, Items N9 and N10 might not be suitable for measuring the physical resilience in hospitalized older patents and could be removed from the PRIFOR.

There was evidence for a 3-factor model with acceptable psychometric qualities. The Rasch analysis also supported the unidimensionality of each factor recommended by the EFA, except for Item N5. The vulnerable or frail older adults included in this study usually suffered from exhaustion. It corresponds to the definition of frailty used to express a multidimensional syndrome of loss of reserves that causes vulnerability [11]. Although physical resilience and frailty are closely related, subtle differences in the two concepts exist (eg, physical resilience has the *recover* concept, whereas *frailty* does not). This might be the reason Item N5 misfit from the Rasch result suggests that item may not deal with the concept of physical resilience and could be removed [4].

The retained 16 items in the PRIFOR all had good psychometric properties and fit in the three-factor structure. The first and third factors are related to the components of psychological resilience, and the second factor exhibits individuals’ strategies for coping and adjusting activities of daily living. The first factor is represented by four items related to positive thinking, such as being able to recover or focusing on individuals’ advantages. The third factor consists of six items addressing belief and hopeful mindset, such as the confidence to handle or deal with an acute health stressor or to have the expectation of a future life. Both factors are related to the components of psychological resilience, which is currently defined as a dynamic ability. Psychological resilience is developed and supported by biopsychosocial and spiritual factors and enable an older adult to spring back and flourish in the face of adversity [31]. Remarkably, a recent study reported psychological resilience associated with recovery of physical function in older adults after hip fracture surgery. Those authors further suggest that interventions targeting psychological resilience may benefit the recovery of older adults with hip fracture [32]. However, psychological resilience is only one factor contributing to a resilient outcome at the whole person level (physical resilience). The remaining second factor consists of six items addressing individuals’ strategies for coping and adjusting their activities of daily living. It corresponds with a new concept of physical resilience, which is not limited to psychological ability, but also extends to the ability to adapt and to self-manage when facing the social, mental, and physical challenges of life [33].

The 16-item PRIFOR had excellent internal consistency and good separation reliability. Additionally, the newly developed PRIFOR has shown good criteria validity. As corresponding to prior evidence [34], there was a significantly negative correlation between the PRIFOR score and depression. Moreover, the PRIFOR score was positively associated with cognitive function, ADL, and quality of life [34–36]. It is worth noting that the known-group validity of the PRIFOR was supported by the significantly higher scores in vulnerable, rather than in frail older adults. Physical resilience and frailty are distinct but related concepts. Whitson et al. (2018) propose that different mechanisms may explain how an individual’s decline in physical condition following a stressor (frailty) and how an individual’s likelihood to counteract or recover from functional loss during and after stressors (physical resilience) [37]. In sum, frailty may refer to any phenotypic function decline after an individual experiences stressor; it may also refer to a substantial and lasting function decline, which further result in loss of phenotypic identity. In each condition, frailty is likely to be synonymous with lack of physical resilience [38].

The present study has some limitations. First, it was conducted in a single medical center in Taiwan, and the results may not be generalizable. Future research is needed to specifically test the reliability and validity of the measure when used across different clinical settings, countries, and cultures. Second, for predicting good physical health outcomes, this study also has not been empirically validated. A longitudinal study is needed to test the predictive validity of the PRIFOR. Third, the present study did not test some important psychometric properties for the PRIFOR; ie, the responsiveness and test–retest reliability of the PRIFOR were not examined. Given that responsiveness and test–retest reliability are important properties for healthcare providers to evaluate the progress of physical resilience and the effectiveness of a treatment program on physical resilience, we encourage future researchers to fill this gap. Fourth, although PRS is not suitable for older adults who suffer from stressors but it seems to be the only subjective measurement of physical resilience. Further studies are needed to include PRS as the criteria validity. Finally, q-sort analysis [39], a powerful analytic method that contains mixed methodology (ie, qualitative and quantitative approaches), was not used in the present study. Therefore, some important perspectives from the target population and stakeholders (ie, older people and healthcare professionals) might not be analyzed for PRIFOR development. Future studies are warranted to use such an analysis to corroborate our PRIFOR psychometric evidence.

### Practical implications

In summary, the concept of physical resilience initiates a paradigm shift for the discipline of healthcare and medicine from disease treatment to building and maintaining strengths required for adaptation of life’s challenges. An adequate estimation of older adults’ level of resilience is crucial. Our study has developed a reliable and valid questionnaire of physical resilience that allows researchers to test intervention effects on resilience improvement. The PRIFOR can assist in identifying older adults who may be at high risk or those who benefit more, before they encounter a particular stressor. It helps in clinical decision making and targeting intensive treatment options (ie, chemotherapy, surgery, or rehabilitation programs) to older adults with the potential to recover would help prevent loss of functional ability. Moreover, this could further aid in developing appropriate care model that could increase resilience and promote recovery to older adults who suffer from stressors.

## Supplementary Information

**Additional file 1.**

**Additional file 2.**

## Data Availability

All relevant data are within the paper and its Supporting Information files.
