# Supplementary material for: Development and psychometric evaluation of the Physical Resilience Instrument for Older Adults (PRIFOR)
Source: BMC Geriatr. 2022 Mar 21;22:229. doi: 10.1186/s12877-022-02918-7 (PMC8935854; doi:10.1186/s12877-022-02918-7)
Supplement: Supplementary file 1 — Additional file 1. [file 12877_2022_2918_MOESM1_ESM.docx]

**Supplementary Material**

Physical Resilience Instrument for Older Adults (PRIFOR)

| Questionnaire | Strongly disagree | Disagree | Agree | Very agree | Strongly agree |
| --- | --- | --- | --- | --- | --- |
| 1. I am able to recover from illness or injury in the expected duration |  |  |  |  |  |
| 1. I believe I can recover from every illness or injury |  |  |  |  |  |
| 1. I try to look on the bright side when I am facing illness or injury |  |  |  |  |  |
| 1. I focus on my remaining abilities, not on what I cannot do |  |  |  |  |  |
| 1. I feel energetic most of the time to do what I have to do |  |  |  |  |  |
| 1. I can cope with the change in my life after illness or injury |  |  |  |  |  |
| 1. I adjust my way of life after illness or injury |  |  |  |  |  |
| 1. When I am ill or injured, I accept help from my families and friends |  |  |  |  |  |
| 1. When I am ill or injured, I accept medical suggestions from healthcare professionals |  |  |  |  |  |
| 1. When I cannot solve a problem, I know where to find help |  |  |  |  |  |
| 1. When I need to, I can find someone to help |  |  |  |  |  |
| 1. I believe I can handle my daily activities |  |  |  |  |  |
| 1. I believe I can recover to do my daily activities after illness or injury |  |  |  |  |  |
| 1. No matter the good or bad things, I believe most of them happen for a reason |  |  |  |  |  |
| 1. Past experience gives me confidence to face new challenges and difficulties |  |  |  |  |  |
| 1. I am a strong person when I am facing illness or injury |  |  |  |  |  |
| 1. I expect and plan for my future life |  |  |  |  |  |
| 1. I can deal with unpleasant or painful feelings like sadness, fear, and anger |  |  |  |  |  |
| 1. I feel I can handle my life |  |  |  |  |  |
| Total score |  | | | | |
